# New Formulation of Platelet-Rich Plasma Enriched in Platelet and Extraplatelet Biomolecules Using Hydrogels

**DOI:** 10.3390/ijms241813811

**Published:** 2023-09-07

**Authors:** Jon Mercader Ruiz, Maider Beitia, Diego Delgado, Pello Sánchez, María Jesús Arnaiz, Leonor López de Dicastillo, Fernando Benito-Lopez, Lourdes Basabe-Desmonts, Mikel Sánchez

**Affiliations:** 1 Arthroscopic Surgery Unit, Hospital Vithas Vitoria, 01008 Vitoria-Gasteiz, Spain; jon.mercader@ucatrauma.com (J.M.R.); pello.sanchez@ucatrauma.com (P.S.); marije.arnaiz@ucatrauma.com (M.J.A.); leonor.lopez@ucatrauma.com (L.L.d.D.); 2 Microfluidics Cluster UPV/EHU, BIOMICs Microfluidics Group, Lascaray Research Center, University of the Basque Country UPV/EHU, 01006 Vitoria-Gasteiz, Spain; 3 Advanced Biological Therapy Unit, Hospital Vithas Vitoria, 01008 Vitoria-Gasteiz, Spain; maider.beitia@ucatrauma.com (M.B.); diego.delgado@ucatrauma.com (D.D.); 4 Microfluidics Cluster UPV/EHU, Analytical Microsystems & Materials for Lab-on-a-Chip (AMMa-LOAC) Group, Analytical Chemistry Department, University of the Basque Country UPV/EHU, 48940 Leioa, Spain; fernando.benito@ehu.eus; 5 Basque Foundation of Science, IKERBASQUE, 48009 Bilbao, Spain

**Keywords:** platelet-rich plasma, hydrogel, water absorption, platelets, biomolecules, growth factors

## Abstract

Platelet-rich plasma (PRP) is an autologous biologic product used in several fields of medicine for tissue repair due to the regenerative capacity of the biomolecules of its formulation. PRP consists of a plasma with a platelet concentration higher than basal levels but with basal levels of any biomolecules present out of the platelets. Plasma contains extraplatelet biomolecules known to enhance its regenerative properties. Therefore, a PRP containing not only a higher concentration of platelets but also a higher concentration of extraplatelet biomolecules that could have a stronger regenerative performance than a standard PRP. Considering this, the aim of this work is to develop a new method to obtain PRP enriched in both platelet and extraplatelet molecules. The method is based on the absorption of the water of the plasma using hydroxyethyl acrylamide (HEAA)-based hydrogels. A plasma fraction obtained from blood, containing the basal levels of platelets and proteins, was placed in contact with the HEAA hydrogel powder to absorb half the volume of the water. The resulting plasma was characterized, and its bioactivity was analyzed in vitro. The novel PRP (nPRP) showed a platelet concentration and platelet derived growth factor (PDGF) levels similar to the standard PRP (sPRP), but the concentration of the extraplatelet growth factors IGF-1 (*p* < 0.0001) and HGF (*p* < 0.001) were significantly increased. Additionally, the cells exposed to the nPRP showed increased cell viability than those exposed to a sPRP in human dermal fibroblasts (*p* < 0.001) and primary chondrocytes (*p* < 0.01). In conclusion, this novel absorption-based method produces a PRP with novel characteristics compared to the standard PRPs, with promising in vitro results that could potentially trigger improved tissue regeneration capacity.

## 1. Introduction

Osteoarthritis (OA) is a degenerative cartilage pathology that currently offers few pharmacological alternatives. In fact, current treatments focus solely on relieving symptoms and not on tissue repair, eventually implying a total knee arthroplasty [1,2]. However, in recent years, biological treatments, such as platelet-rich plasma (PRP), have shown efficacy in managing these cartilage lesions, improving symptomatology, promoting tissue regeneration, and significantly delaying the need for joint replacements [3].

Platelet-rich plasma, also known as PRP, has emerged as a therapeutic approach in numerous medical areas, including orthopedics and traumatology [4,5], dermatology [6,7,8], and oral and maxillofacial surgery [9], due to its high concentration of growth factors (GFs) that promote tissue regeneration and wound healing.

PRP is obtained by concentrating platelets from autologous blood [10]. Platelets are anucleate subcellular fragments with a life span of 7 to 10 days and present in blood at a concentration of 150,000–450,000 platelets μL^−1^. They play a crucial role in wound healing and tissue regeneration due to their ability to release numerous bioactive molecules, including platelet-derived and plasma extraplatelet GFs, upon activation [10,11]. The GF inside the platelets, including the platelet-derived growth factor (PDGF), transforming growth factor-beta (TGF-β), and vascular endothelial growth factor (VEGF), among others, are stored in platelet alpha granules [12]. These GFs have been shown to stimulate cell proliferation, the chemotaxis stimulation of macrophages and neutrophils, angiogenesis and vasculogenesis, extracellular matrix synthesis, and tissue regeneration, making them key players in the regenerative properties of PRP [13,14,15,16,17,18,19].

Hepatocyte growth factor (HGF) and insulin-like growth factor (IGF-1) are considered extraplatelet molecules, as they are in a higher concentration in the plasma than inside alpha granules [20,21]. These extraplatelet GFs promote cell growth, proliferation, migration, and differentiation capacity together with a high anti-inflammatory and antifibrotic effect [20,22,23,24], making them a key factor in tissue regeneration and wound healing processes as well. On the one hand, IGF-1 has been extensively studied in various regenerative medicine applications, including musculoskeletal injuries, skin wound healing, and tissue engineering. Studies have shown that an increase in IGF-1 in PRP formulation can enhance its regenerative properties [25,26]. On the other hand, in recent years it has been shown that HGF has a very potent anti-inflammatory effect [23,27], which would make PRP treatment much more effective. The concentration of these biomolecules in plasma is not dependent on platelet concentration [28].

The concentration and composition of GFs in the PRP can vary depending on various factors, including the PRP preparation method, platelet activation status, and patient characteristics [29], which may affect the therapeutic efficacy of PRP in different clinical applications. One of the key steps in the preparation of PRP is the separation of platelets from other blood components, which usually involves centrifugation. However, these conventional methods for PRP preparation may suffer from limitations such as low platelet recovery, inconsistent GF concentrations, and the inability to enrich plasma proteins. A potential option to concentrate also plasma proteins is the application of a system that removes water and concentrates all plasma components equally.

In recent years, hydrogels have gained significant attention due to their properties such as biocompatibility, high water absorption capacity, and tunable mechanical properties [30]. They are three-dimensional networks of crosslinked polymers that can absorb large amounts of water [31]. Among all the great variety of hydrogels and the properties provided by each one, the hydroxyethyl acrylamide (HEAA) monomer has proven to have a great capacity to absorb water and, above all, to repel proteins and prevent them from sticking to the surface of the material. This condition is given due to the formation of a hydration layer on the surface of the gel [32,33]. The water molecules bound with the polymer layer form a physical and energetic barrier that hinders protein adsorption [34]. These properties make them an option for the removal of water from plasma without causing the loss of other components.

Considering all the above mentioned, our hypothesis is that the elimination of the water in the plasma would allow the concentration of all the intra- as well as extraplaquetary components, which could improve the bioactivity of the new PRP.

Accordingly, the aim of this work is to develop a new technique for the obtention of a novel PRP based on the absorption of water via hydrogels for an improved enrichment of the components of the PRP. Additionally, we aim to characterize the resulting nPRP in terms of cellular and molecular content, platelet activation levels, the capacity to generate a clot, and the in vitro capacity to boos cellular proliferation.

## 2. Results

### 2.1. Water Absorption Parameters Optimization for nPRP Obtention

**Figure 1:**
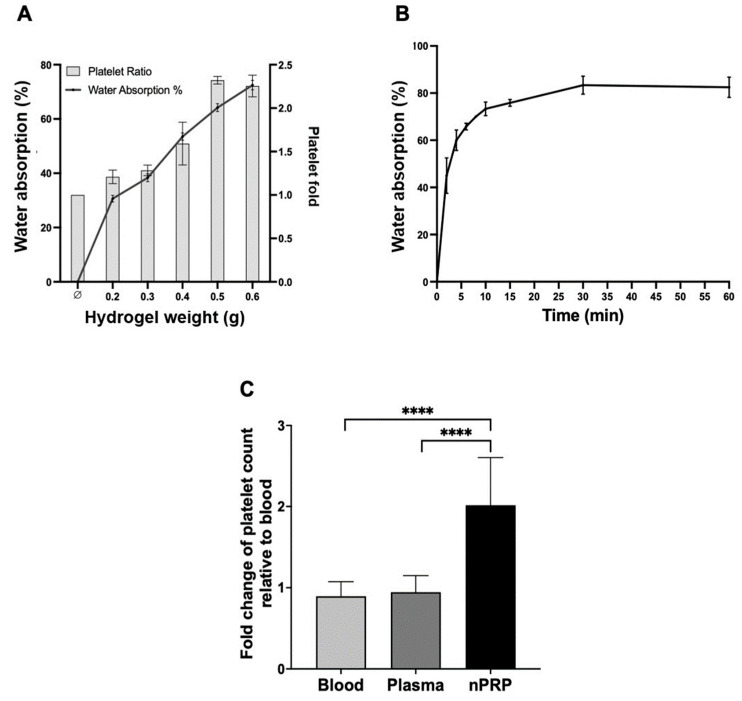
Water absorption and platelet concentration capacity of hydroxyethyl acrylamide (HEAA) hydrogel. (**A**) Plot of water % absorption and platelet concentration upon exposure of 4 mL of plasma samples to different weights of hydrogels ranging from 0 to 0.6 g in a specific time set in 5 min. (**B**) Water absorption capacity (%) of 0.4 g of HEAA hydrogel in relation to time. (**C**) Comparison of blood, the plasma obtained after the centrifugation and the novel PRP (nPRP) obtained after hydrogel exposure, in terms of platelet content. Error bars = standard deviation (*n* = 8). Statistically significant differences were calculated using one-way ANOVA (**** *p* < 0.0001).

To establish the parameters of the hydrogel absorption process to remove half of the volume of water from the plasma, the required amount of hydrogels and the absorption time were established. The hydrogel amount used ranged from 0.2 to 0.6 g for an initial plasma volume of 4 mL, and an absorption time of 5 min was set. Under these conditions, the amount of water absorbed, by measuring the initial and final plasma volume, and the resulting platelet concentration were measured. The results showed an increase in water absorption and platelet concentration as the weight of the hydrogel increased (Figure 1A). Thus, it was established that the amount of hydrogel needed to absorb half the water and double the platelet concentration was 0.4 g per 4 mL of plasma (ratio of 1:8).

Furthermore, an absorption curve was generated to determine the absorption capacity of the hydrogels over time. A progressive increase in water absorption over time until it reached a plateau was observed. At 5 min, the slope of the curve decreases, and after 30 min, it becomes saturated. Therefore, the time chosen for maximum water absorption was 5 min (Figure 1B).

The concentration of platelets was characterized in samples processed only via centrifugation and samples processed via centrifugation and hydrogel exposure. The hydrogel-exposed samples had double the amount of platelets compared to initial levels in whole blood and compared to the sample just processed via centrifugation (*p* < 0.0001 for both cases). This observation is in agreement with an absorption of half of the volume of water in the plasma by the hydrogel (Figure 1C).

### 2.2. Comparison of Platelets and Total Proteins Concentration Capacity between a Standard (sPRP) and a Novel (nPRP) PRP

**Figure 2:**
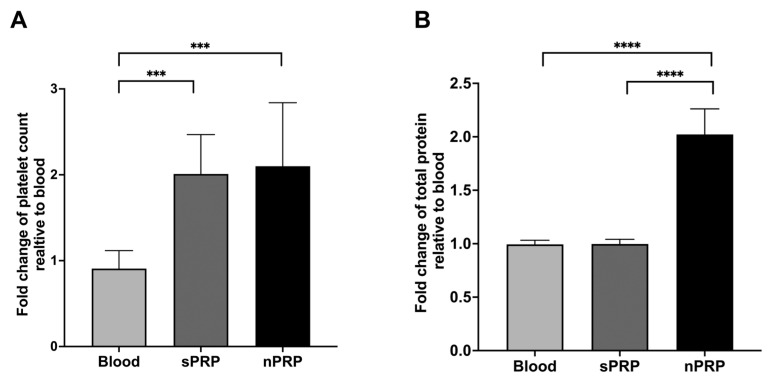
Platelet and total protein concentration levels in standard and novel PRP. Mean values of platelet (**A**) and protein (**B**) in blood and standard and novel PRP are shown. Error bars = standard deviation (*n* = 8). Statistically significant differences were calculated using Kruskal–Wallis for the platelet count and one-way ANOVA for the total protein levels (*** *p* < 0.001; **** *p* < 0.0001).

The composition of the nPRP obtained using sequential centrifugation and hydrogel exposure was compared to the composition of blood and standard PRP (sPRP). The results showed that the platelet content in both plasmas (standard and novel) was twice as high as in blood, and these differences were statistically significant (*p* = 0.0004 and *p* = 0.0007, respectively). However, no statistically significant differences were found between sPRP and nPRP (*p* > 0.9999) (Figure 2A).

In terms of the total protein content, nPRP showed twice the total protein amount than that found in blood and sPRP (*p* > 0.0001 for both cases). There were no significant differences between blood and sPRP (*p* = 0.9827) (Figure 2B).

### 2.3. Concentration of Growth Factors in sPRP and nPRP

**Figure 3:**
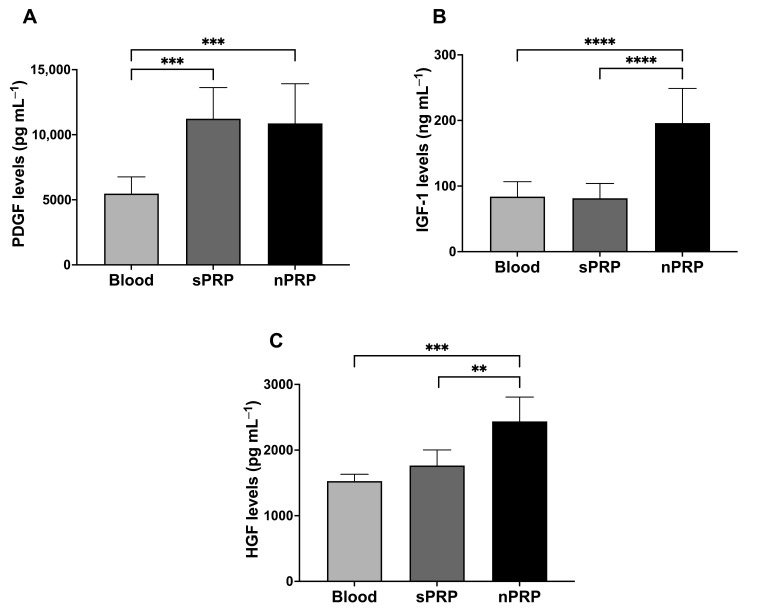
Platelet and extraplatelet growth factor levels. Mean values of platelet growth factor PDGF (**A**), the extra-platelet IGF-1 growth factor (**B**), and the intra- and extra-platelet growth factor HGF (**C**) of blood and standard and novel PRP are shown. Error bars = standard deviation (*n* = 8). Statistically significant differences were calculated using one-way ANOVA (** *p* < 0.01; *** *p* < 0.001; **** *p* < 0.0001).

In order to evaluate the plasma protein enrichment in nPRP, three different proteins were measured: IGF-1, an extraplatelet growth factor that is not present inside platelets, HGF, which is found both inside and outside platelets (although predominantly outside), and PDGF, which is found exclusively inside platelets. The results showed that both sPRP and nPRP contained double the amount of the platelet growth factor PDGF compared to basal levels in the blood (*p* = 0.0003 and *p* = 0.0002, respectively), and no statistical differences were found between the two PRPs (*p* = 0.9226) (Figure 3A). In the case of extraplatelet factor IGF-1, it was concentrated two-fold in nPRP but was not enriched in sPRP compared to basal levels in the blood (*p* < 0.0001 in both cases). No differences were found between blood and sPRP (*p* = 0.8205) (Figure 3B). Finally, when measuring the concentration of HGF, nPRP contained significantly higher levels compared to basal levels in the blood and to sPRP (*p* = 0.0004 and *p* = 0.0013, respectively). However, the concentration of HGF did not increase in sPRP compared to basal levels in blood (*p* = 0.0542) (Figure 3C).

### 2.4. Ion Concentration Capacity of sPRP and nPRP

**Figure 4:**
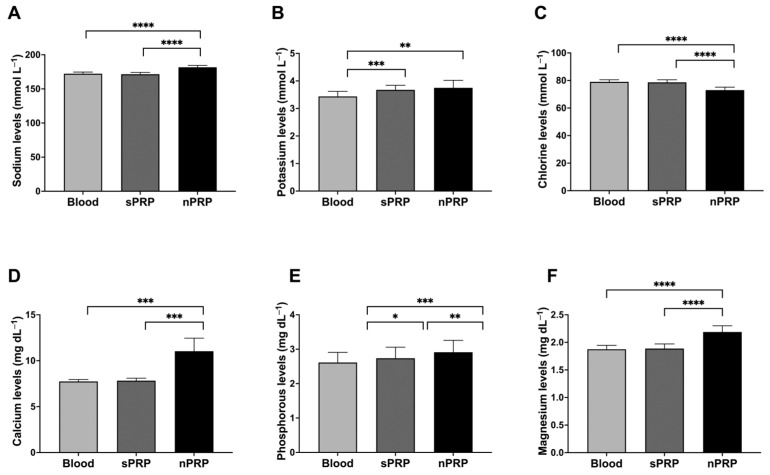
Ion concentration of the standard and novel PRP. Mean values of sodium (**A**), potassium (**B**), chlorine (**C**), calcium (**D**), phosphorous (**E**), and magnesium (**F**) are shown. Error bars = standard deviation (*n* = 8). Statistically significant differences were calculated using one-way ANOVA (* *p* < 0.05; ** *p* < 0.01; *** *p* < 0.001; **** *p* < 0.0001).

In order to analyze hydrogel absorption of salts, the levels of sodium (Figure 4A), potassium (Figure 4B), chlorine (Figure 4C), calcium (Figure 4D), phosphorus (Figure 4E), and magnesium (Figure 4F) ions were measured in blood, sPRP, and nPRP. The results showed a slight but statistically significant increase in the concentration of salts in nPRP compared to sPRP and whole blood, except in the case of chlorine, where the levels decreased. However, in cases such as potassium and phosphorus, these differences were also observed between sPRP and blood.

### 2.5. sPRP and nPRP Processing Impact on Platelet Activation

**Figure 5:**
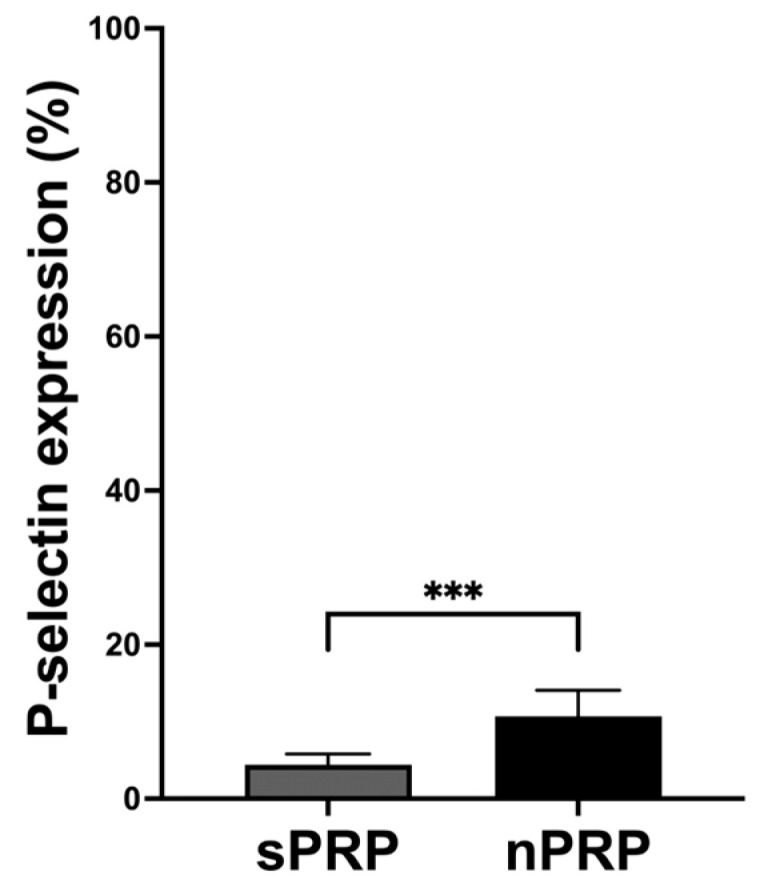
Platelet activation in standard and novel PRP. The graph represents the CD62-positive cells that are indicative of activated platelets. Error bars = standard deviation (*n* = 8). Statistically significant differences were calculated using *t*-test (*** *p* < 0.001).

In addition, the level of platelet activation produced during the plasma obtention was evaluated. The analysis was based on the measurement of CD41 (platelet marker) and CD62 or p-selectin (a marker of activated platelets). The results showed statistically a significantly increased activation of platelets in nPRP samples compared to the activation in sPRP samples (*p* < 0.001) (Figure 5).

### 2.6. Fibrinogen Levels and Biomechanical Properties of the Clot Resulting from sPRP and nPRP

**Figure 6:**
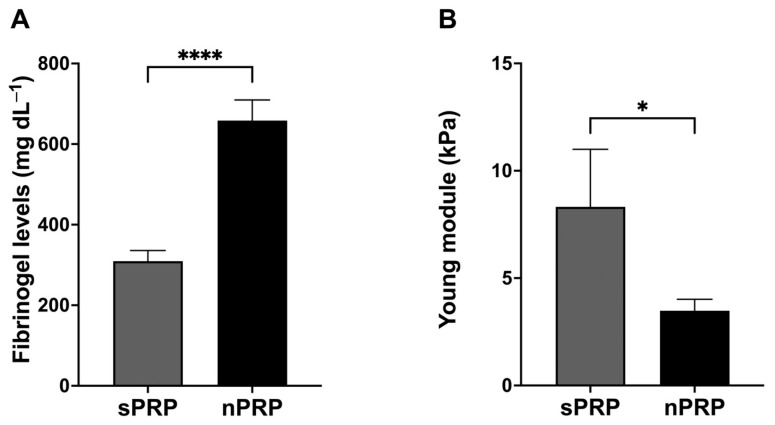
Fibrinogen levels and clot properties of the standard and novel PRP. Mean values of fibrinogen levels (**A**) and Young’s module of the fibrin clot (**B**) of the standard and novel PRP are shown. Error bars = standard deviation (*n* = 8). Statistically significant differences were calculated using *t*-test (* *p* < 0.05; **** *p* < 0.0001).

During platelet activation, the fibrin clot formed in sPRP and nPRP formulations showed differences in terms of clot retraction. The clot formed in sPRP was much softer than the one obtained via nPRP, and it was retracted completely unlike the nPRP clot. Hence, fibrinogen values and Young’s modulus of both formulations were analyzed. On the other hand, in order to analyze the possible differences that might exist between the clots formed from each type of PRP, the concentrated fibrinogen was measured in each of the samples. The results showed that the concentration of fibrinogen was significantly higher in nPRP than in sPRP (*p* < 0.0001) (Figure 6A).

In addition, Young’s modulus measurements were performed to test for biomechanical differences between the two clot types. It was found that the nPRP clot had a lower Young’s modulus than sPRP, indicating lower stiffness and, therefore, higher elasticity (*p* < 0.05) (Figure 6B).

### 2.7. Viability of Fibroblasts Culture upon Exposure to nPRP

**Figure 7:**
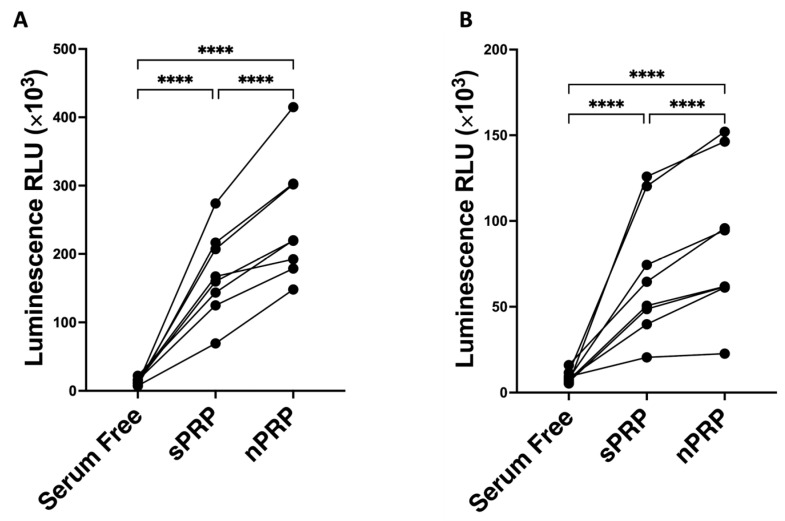
Cellular viability in NHDF cells and human primary chondrocytes. The viability levels of (**A**) NHDF cells and (**B**) human primary chondrocytes incubated with standard and novel PRP are expressed as relative light units (RLUs), and each point represents a different donor (*n* = 8). Serum-free conditions served as negative control. Statistical analysis was calculated using one-way ANOVA (**** *p* < 0.0001).

Finally, the bioactivity of each PRP was assessed by measuring its ability to promote cell proliferation in dermal fibroblasts and primary chondrocytes. To do so, these cells were incubated in the presence of either sPRP or nPRP for 96 h, after which a luminescence-based technique was used to determine viability at that time. The results showed that cells incubated with nPRP presented higher levels of viability compared to those incubated with sPRP (*p* < 0.001) (Figure 7A). Concerning the primary chondrocytes, an increase in the viability of the cells incubated with nPRP compared to those incubated with sPRP was observed (*p* < 0.01) (Figure 7B).

## 3. Discussion

In this present work, a method was developed to obtain a PRP enriched in both platelet and extraplatelet biomolecules based on the use of acrylamide hydrogels. This hydrogel was developed by Zhao et al., which, in turn, was based on another work conducted by the same author where they characterized it in terms of physical stability, swelling behavior, size, composition, anti-fouling ability, and water uptake, among others [35,36,37]. In addition, the washing procedure carried out after its fabrication ensured the total elimination of residual compounds based on a work conducted by Gertsiuk et al. [38]. Moreover, the biosafety of HEAA-based hydrogels was tested by Peng et al. In that study, they performed a cell viability assay and a hemocompatibility test showing high biocompatibility [39]. The developed method produced PRP with a platelet concentration that doubled the levels in the blood, similar to conventional PRP products. Moreover, and in contrast to the common obtaining methods, the PRP achieved in this work also showed an enrichment of extraplatelet biomolecules due to the absorption of water in the hydrogels and the consequent concentration of plasma content. Additionally, the novel PRP showed similar levels of the platelet factor PDGF as the sPRP but significantly increased extraplatelet IGF-1 and HGF. Although ion concentration and activation were slightly increased, cells showed a better response in terms of cell viability with nPRP than those exposed to sPRP.

One of the reasons to use this hydrogel in the production of PRP is its ability to absorb water from the plasma but not adhere to proteins or cells. HEAA is a polymer with properties that were studied previously due to its antifouling capabilities. This ability to not adhere to proteins or cells is due to two factors: the hydration layer generated by its hydrogen bonds and the charge–charge interaction. Thus, the HEAA hydrogel has a neutral charge, so there is no electrostatic interaction between the protein and the hydrogel. It is, therefore, considered to be one of the biomaterials with the highest antifouling capacity [1,2,3,4,5,6]. In fact, the results showed an absorption of half the volume of the plasma water in the first 5 min after putting the hydrogel in contact with the plasma. This is a competitive time for obtaining the PRP in comparison with other commercially available systems, which have processing times of between 5 and 15 min approximately [40]. In addition to the water absorption capacity, the use of HEAA provides the hydrogel with a high antifouling capacity that avoids the attachment of proteins and other plasma molecules to the developed hydrogel [35] due to the water molecules around the structure of the gel that generates a hydration layer that gives the antifouling capacity [32,33,34]. In this way, the entire plasma content, including platelets, is present in the final product. Therefore, through this method, a PRP in which both platelets and extraplatelet biomolecules are equally concentrated is obtained. In the particular case of this work, the concentration is double the blood levels by absorbing half the volume of water. However, this concentration could be modulated by varying the amount of hydrogel used or the contact time.

Considering that the effects of PRP are in part due to its modulation of biological processes [10], an improvement in these growth factor-mediated processes could be translated into better clinical outcomes. Indeed, it has been shown that plasma factors such as IGF-1 are key factors in cellular processes involved in the mechanism of action of PRP [20,28,41]. Moreover, this could be beneficial in elderly patients as this GF decreases with age [42]. In line with these studies, the findings in this work showed that the modification of the molecular composition, i.e., increasing the levels of extraplatelet molecules, enhanced the bioactivity of the new PRP compared to the conventional one since fibroblasts cultured in the presence of nPRP showed higher cell proliferation at 96h compared to those cultured with sPRP.

The variation in salt levels could be related to an alteration in clot formation after PPR activation [43,44,45]. No difference in coagulation time was observed in nPRP after the addition of the activator CaCl_2_ compared to sPRP. However, the clot formed from nPRP exhibited higher elasticity and, thus, lower stiffness than the clot from the standard PRP, probably caused by an increase in the fibrinogen concentration. These new biomechanical properties could be favorable for the application of the clot in surgical applications [46].

One of the possible limitations of the hydrogel-based PRP process could be the increased manipulation of blood and the effects of hydrogels on the platelets due to the interaction between the different components. However, after analyzing platelet activation, it was observed that although it was higher than in the centrifugation standard process, this platelet activation was still not influential either in obtaining the PRP or in the properties of the final product.

Overall, the nPRP formulation obtained using this hydrogel-based method, which concentrates extraplatelet molecules in addition to platelet-derived ones, has a positive impact on in vitro cell cultures. This was confirmed in both NHDF and primary chondrocytes. This could imply an increased efficiency in tissue regeneration for both skin and cartilage lesions. Osteoarthritis affects millions of people worldwide. With only a few disease-modifying drugs available for the treatment of rheumatoid arthritis and none for osteoarthritis, a clear need exists for new treatment options such as the one proposed in this work [47]. However, it would be interesting to explore other tissues as well. Furthermore, and importantly, the results obtained suggest that these hydrogels are potentially inert in their interaction, avoiding any alteration in the PRP or possible cytotoxicity, which would guarantee their biosafety.

The main limitation of this work is the limited number of processes analyzed in the in vitro part of this study. In fact, only cell viability was measured. In further studies, other features of the nPRP, such as its anti-inflammatory capacity via the analysis of the released interleukins, cell migration capacity via a scratch assay, or the analysis of differentiation markers, among others, should be considered. Future studies analyzing larger sample sizes and using organ-on-chip models would help the understanding of the potential of this new product. Additionally, further characterization of the employed hydrogel has not been carried out and would be of interest to future studies. Although the washes performed are adequate to remove all acrylamide components according to one study [38], it would be interesting to perform a chromatographic analysis for the detection of free acrylamide monomers as well as biosafety tests. It will be also necessary to verify that the increase in biomolecule concentration on the nPRP is able to generate real clinical improvements in patients.

## 4. Materials and Methods

### 4.1. Donors

Eight healthy donors were selected ranging in age from 29 to 58 years old (4 female/4 male). Whole blood was withdrawn into tubes of 9 mL and 3.5 mL containing 3.8% (*w*/*v*) sodium citrate. Additionally, 3.5 mL tubes were used to measure platelet concentration at baseline levels, while the others were used to obtain the sPRP and the nPRP. Ethical approval was obtained from Ethics Committee of UPV/EHU (2019-234, 13 May 2020), and written consent was obtained from patients.

### 4.2. Fabrication of the Hydroxyethyl Acrylamide (HEAA)-Based Hydrogel Powder

Fabrication of the HEAA monomer was carried out as described by Chao Zhao et al. [35] For the fabrication of the HEEA hydrogel, 4 mM of HEAA monomer, 10 mg of photoinitiator 2-hydroxy-4′-(2-hydroxyethoxy)-2-methylpropiophenone, and 2.5% of N,N′-methylenebisacrylamide (MBAA) (all from Sigma-Aldrich, Saint Louis, MO, USA) as cross-linkers were added, per 1.5 mL of solution. Total volumes used for the formulation were 0.375 mL of ethanol, 0.565 mL of ethylene glycol, and 0.565 mL of distilled water (DH_2_O). The resulting solution was gently mixed at RT for 1 h until all the reagents were completely dissolved. To polymerize it, the solution was placed in a 100 mL beaker. Polymerization was initiated at room temperature with a 365 nm UV light (Thermo Scientific 3UV lamp, Fisher Scientific, Waltham, MA, USA) for 1 h, turning the gel upside down after 30 min to favor the polymerization of the inner part not directly exposed to UV light. The resulting hydrogel block of 55 × 60 mm was then removed from the beaker and crushed by passing it through a manual mortar until small pieces of approximately 2 × 2 mm were obtained. After that, hydrogel particles were soaked in deionized water for 7 days. The water was changed three times per day to ensure that no unreacted chemicals, especially acrylamide, remained in the sample, and it was completely removed from the hydrogels [38].

After the washing step, the hydrogels were dried in an oven for 24 h at 56 °C. After dehydration, they were crushed again in a mortar, and the dried hydrogel pieces were passed through a platform consisting of 3 sieve shakers ranging in size from 1 to 0.5 mm for hydrogel particle separation. The desired particle size was selected to be between 0.5 and 1 mm to obtain hydrogel particles with a controlled homogeneous size.

### 4.3. Standard Platelet-Rich Plasma (sPRP) Preparation

**Figure 8:**
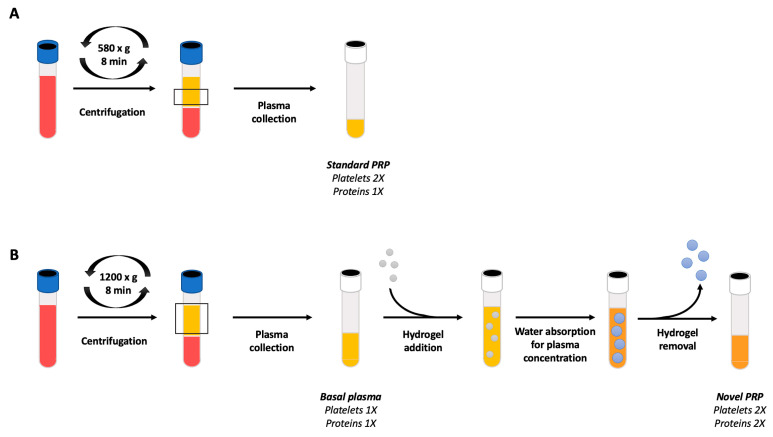
PRP obtaining process. (**A**) Schematic representation of the process to obtain sPRP: 2 mL plasma above the red blood cell fraction is collected from an initial volume of 9 mL of whole blood, with expected doubled concentration of platelets and total proteins. (**B**) Schematic representation of the process to obtain nPRP using 0.5–1 mm size hydrogel particles. A basal plasma fraction is obtained with expected similar levels of platelets and total protein as in blood (1×). After water absorption and separation of the hydrogel from the concentrated plasma, the final product obtained is a PRP expected to double its concentration of extraplatelet biomolecules and platelets (2×).

The sPRP was obtained via centrifugation of 9 mL of blood at 580× *g* for 8 min at room temperature to obtain the sPRP fraction, after collecting 2 mL of plasma fraction present over the red blood cell fraction (avoiding collecting white blood cells from the buffy coat) (Figure 8A). 10% CaCl_2_ (20 µL mL^−1^) was added to the plasma to trigger platelet activation and clot formation.

### 4.4. Novel Platelet-Rich Plasma (nPRP) Preparation

The nPRP (Figure 8B) was obtained via centrifugation of 9 mL of whole blood at 1200× *g* for 8 min at room temperature, pipetting out the 2 mL basal plasma fraction from each tube, with no red or white blood cells. These centrifugation parameters were set to achieve a basal plasma fraction with both platelet and total protein levels similar to those obtained in whole blood to ensure that the final concentration of the nPRP would double their basal levels.

For the concentration of the platelets and biomolecules of the plasma sample, hydrogel powder ranging from 0.5 to 1 mm in size was loaded into a syringe together with the basal plasma fraction at 0.125 g mL−^1^. The water absorption was performed for 5 min and poured over a 100 µm cell strainer filter (Biologix, Pleasant Prairie, WI, USA) placed in a 50 mL tube. The tube was centrifuged at 500× *g* for 2 min to separate the concentrated plasma from all the hydrated hydrogel particles.

### 4.5. Platelet and Protein Fold Measurement

Platelet and protein concentration levels were measured in whole blood, sPRP, and nPRP samples from all the donors. Platelets were measured with a hematology analyzer (Sysmex XS-1000i; Kobe, Japan), whereas total protein levels were measured using a Cobas c 501 analyzer (Roche, Basel, Switzerland).

### 4.6. Platelet and Plasmatic Growth Factor Concentration Measurement

Both platelet and plasmatic GFs, such as IGF-1, HGF, and PDGF-AB, present in sPRP and nPRP were analyzed as follows. First, plasma samples were activated by adding 10% CaCl_2_, and then the concentration of those GFs was measured using commercially available enzyme-linked immunosorbent assay (ELISA) kits (R&D Systems, Minneapolis, MN, USA).

### 4.7. Ion Levels Measurement

Ion concentration measurements were carried out for whole blood, sPRP, and nPRP plasma samples using a Cobas c 501 analyzer (Roche, Basel, Switzerland). Ca^2+^, Na^+^, Cl^−^, Mg^2+^, P^3−^, and K^+^ were the selected ions to be quantified.

### 4.8. Platelet Activation Test

Measurement of p-selectin was used as a method to test the platelet activation. P-selectin, also called CD62P, is stored in α-granules of inactivated platelets. After platelet activation, the inner walls of these granules are exposed on the outside of the cells, presenting the CD62P protein [48]. For that purpose, CD41 and CD62P antibodies, which recognize platelet membrane constitutive glycoprotein GpIIb and translocated p-selectin, respectively, were used.

For each sample, 10 μL of plasma sample, 5 μL of anti-CD41-FITC, and 5 μL of anti-CD62P-PE (BD Biosciences, San Jose, CA, USA) were added in the test tubes. The remaining volume up to 100 μL was completed with PBS. Finally, samples were incubated for 15 min at RT in the dark and then were fixed with 400 μL of freshly prepared 1.25% formaldehyde (PanReac AppliChem, Barcelona, Spain) in PBS (Gibco, Waltham, MA, USA). A Gallios flow cytometer (Beckman-Coulter, High Wycombe, UK) was used to analyze samples.

### 4.9. Fibrinogen Levels Measurements and Biomechanical Tests on Fibrin Clots

Fibrinogen levels were measured using the coagulation analyzer (STA Compact Max, Stago, France).

To determine the biomechanical properties of the fibrin clots, instrumented indentation tests were carried out using a spherical indenter. A calibrated spherical indenter with a diameter of 5 mm was pressed onto the sample, and the load used to penetrate the indenter as well as the penetration distance at each moment were recorded. The sphere penetrated the sample at a constant speed of 50 μms^−1^ until it reached a depth equivalent to 20% of the initial thickness of the sample. Once this penetration was reached, the direction of movement was reversed, discharging the indenter. Young’s modulus and dissipated energy were analyzed. All tests were performed with a Zwick/Roell ZwickiLine Z1.0 uniaxial testing machine (Ulm, Germany). The load cell used was a Zwick/Roell Xforce P with a maximum load of 50 N.

### 4.10. Normal Human Dermal Fibroblast (NHDF) Cell Cultures

Normal human dermal fibroblast (NHDF) (Lonza, Basel, Switzerland) were kept in the incubator at 37 °C and 5% CO_2_ atmosphere. Cells were grown in fibroblast growth basal medium (FBM, Lonza, Basel, Switzerland) supplemented with insulin, human fibroblast growth factor, and gentamicin sulfate-amphotericin at 0.1% (*v*/*v*) each (Lonza, Basel, Switzerland), as recommended by the manufacturer.

### 4.11. Isolation and Culture of Human Primary Chondrocytes

Chondrocytes were isolated from the knee condyles and tibial plateau during a total knee replacement surgery. The cartilage pieces were immersed and washed three times in 1 × PBS with 1% penicillin-streptomycin. Then, the cartilage was cut into 2 × 2 mm pieces. A solution composed of Hanks’ Balanced Salt Solution (HBSS) (Gibco, Waltham, MA, USA) and collagenase II (Gibco, MT, USA) at 0.1–0.2% was added per gram of cartilage and incubated overnight at 37 °C and 5% CO_2_. The following day, the cartilage pieces were filtered through a 40 µm cell strainer so that the remnants of the extracellular matrix of the cartilage remained on the surface of the filter, and the HBSS with the cells fell into the falcon. Two washes with PBS were carried out via centrifugation at 300× *g* for 10 min. Then, the cellular pellet was resuspended in high glucose and L-glutamine-containing Dulbecco’s Modified Eagle Medium (DMEM) (ATCC, Manassas, VA, USA) + 10% FBS + P/S and placed in a T25 flask. Chondrocytes were used in passage 2 for the experimental analyses [49].

### 4.12. Cell Viability Assay

For the evaluation of the biological activity of both sPRP and nPRP, NHDF were incubated with FBM medium supplemented with 10% of either sPRP or nPRP platelet lysates, and cellular viability was registered at 96 h. Cellular viability was measured in triplicate for each donor via a Realtime-Glo MT Cell Viability Assay (Promega, Fitchburg, MA, USA) based on the reducing potential of metabolically active cells that catalyze the conversion of a synthetic substrate into a luminescent product. Luminescence reading was performed via a TECAN Infinite 200 PRO plate reader (TECAN, Zurich, Switzerland). The level of luminescence can be considered proportional to the number of viable cells present in the assay [50].

### 4.13. Statistical Analysis

Distribution of the samples was assessed using Shapiro–Wilk’s normality test. The different variables were determined via the mean and the standard deviation for parametric data. Comparisons were performed using ANOVA and Student’s *t*-test. Data were considered statistically significant when *p* < 0.05. GraphPad Prism^®^ software version 9.5 (San Diego, CA, USA) was used for the statistical analysis.

## 5. Conclusions

Overall, this novel technique’s main feature is the ability to concentrate extraplatelet molecules in addition to platelets, which has a positive impact on its bioactivity and the formation of the fibrin matrix. Furthermore, importantly, these results suggest that these hydrogels are potentially inert in their interaction with PRP, avoiding possible cytotoxicity, which would guarantee their biosafety.

Given that this sPRP has a balance of platelets and biomolecules, unlike sPRP, and with a greater number of plasma molecules, its biological and therapeutic effect could be more efficient in the field of regenerative medicine, potentially improving the clinical outcome of patients.

## Data Availability

No new data were created.
